# Algorithm and ninhydrin method allow for measurement of the postprandial appearance of peptides in blood

**DOI:** 10.1038/s41598-025-04625-w

**Published:** 2025-06-04

**Authors:** Stefan G. Pierzynowski, Piotr Wychowański, Wiesław Szczesny, Robert Galloto, Kamil Zaworski, Dominika Szkopek, Jarosław Woliński, Janine Donaldson, Kateryna Pierzynowska

**Affiliations:** 1 Department of Biology, Lund University, Lund, Sweden; 2 Anara AB, Trelleborg, Sweden; 3 Department of Medical Biology, Institute of Rural Health, Lublin, Poland; 4 Department of Head and Neck and Sensory Organs, Division of Oral Surgery and Implantology, Institute of Clinical Dentistry, Gemelli Foundation for the University Policlinic, Catholic University of the “Sacred Heart”, Rome, Italy; 5 Department of Interventional Dentistry, Collegium Medicum, Nicolaus Copernicus University, Bydgoszcz, Poland; 6 Specialized Private Implantology Clinic Wychowański Stomatologia, Warsaw, Poland; 7 Institute of Information Technology, Warsaw University of Life Sciences, Warsaw, Poland; 8 Anagram Therapeutics, Inc, Natick, MA USA; 9 Department of Animal Physiology, The Kielanowski Institute of Animal Physiology and Nutrition, Polish Academy of Sciences, Jabłonna, Poland; 10 Large Animal Models Laboratory, The Kielanowski Institute of Animal Physiology and Nutrition, Polish Academy of Sciences, Jabłonna, Poland; 11 School of Physiology, Faculty of Health Sciences, University of the Witwatersrand, Parktown, Johannesburg, South Africa

**Keywords:** Revisited ninhydrin method, Algorithm, Protein digestion end products, Dipeptides, Tripeptides, Mixed meal tolerance test, Gastrointestinal models, Homeostasis, Diagnostic markers

## Abstract

The recognition of amine groups by ninhydrin, along with a simple mathematical algorithm, showed that di- and tripeptides derived from dietary protein are the major end products of protein digestion entering the blood postprandially. There are thousands of oligopeptides appearing in the gut during protein digestion. However, the presented study on a pig model clearly shows that peptides longer than tri-amino acid peptides do not appear postprandially in the blood in nutritional amounts. We hypothesize that the measurement of postprandial free amino acids, di- and tripeptides and proportions between these components could be a useful, desirable tool both in laboratory and clinical practice, which could be used to determine the metabolic importance of protein digestion end products in health and disease.

## Introduction

The blood appearance of protein digestion end products (free amino acids and peptides) and the ratio in which they appear in the blood postprandially is poorly understood. Elegant, sophisticated and expensive methods (HPLC, MS, MS–LC, etc.) are inadequate when estimating the number of amino acids coming from the absorbed peptides^1^. Results from the same laboratory, obtained using different methods, sometimes differ by more than 100%^1^. Recently^2,3^ a revisited ninhydrin method has successfully been used for the assessment of the postprandial presence of amine groups in the blood of healthy and exocrine pancreatic insufficient (EPI) pigs, as well as in cystic fibrosis (CF) patients.

**Fig. 1:**
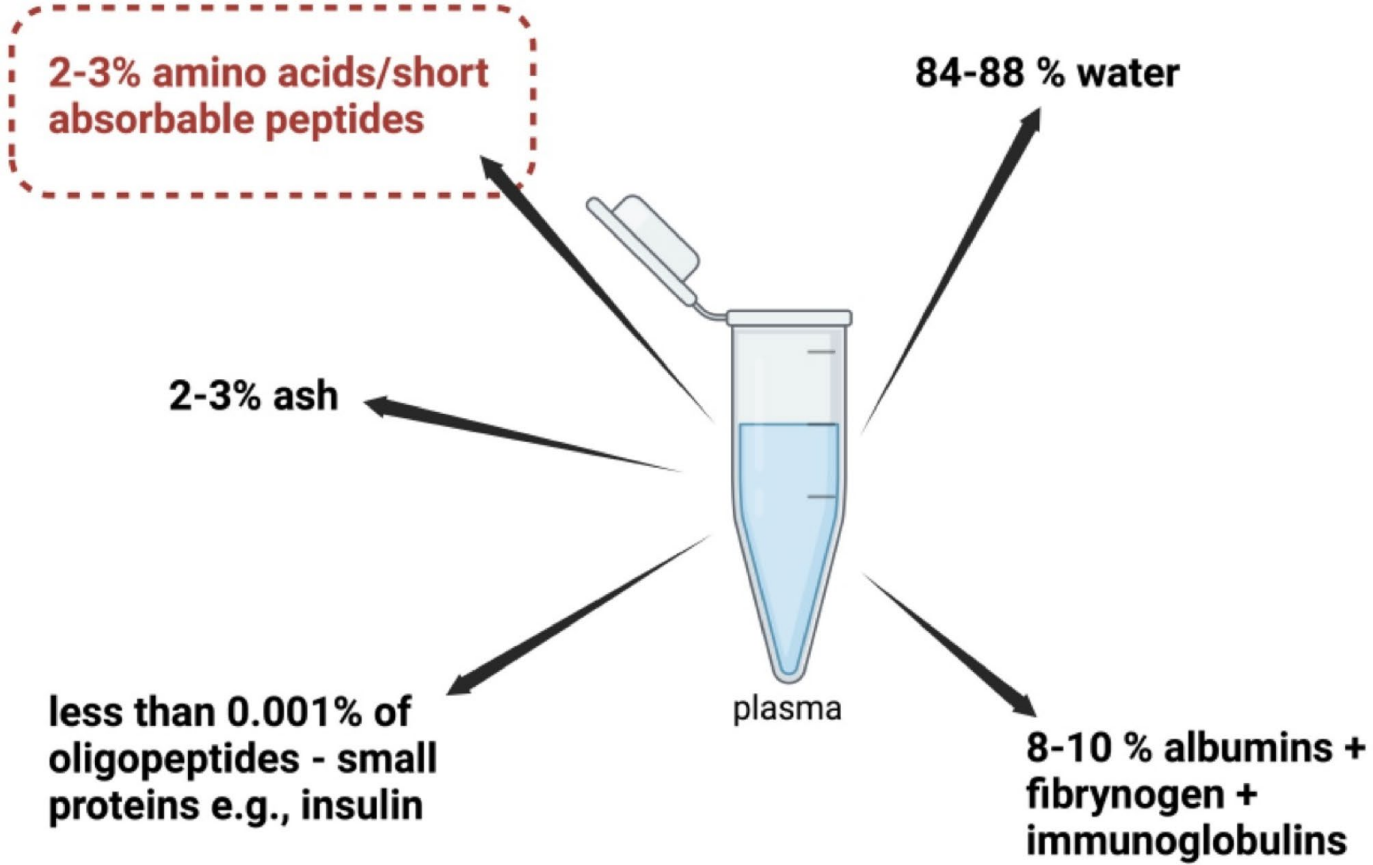
Schematic diagram of blood plasma/serum composition.

The understanding of the differences in peptide versus amino acid uptake served as the basis for the development of the algorithm. However, to understand the algorithm principles it is necessary to refresh some crucial basic knowledge of blood composition and protein structure. It is common knowledge that serum/plasma consists of ca. 8–10% albumin and other proteins bigger than 60 kD^4^. Free amino acids, together with oligopeptides account for between 2 and 3%^5^ of plasma or serum and they are measured in µg/L or µmol/L, ash accounts for 2–3% and water for around 90%^6^. Oligopeptides bigger than tripeptides in plasma or serum, are generally recognised as bioactive peptides or hormones, for e.g., CCK-4 (4 amino acids) and insulin (51 amino acids). In total they (oligopeptides) account for only an extremely small portion of the blood peptides—far less than 0.001%, since those bioactive peptides are measured in pg/L, while peptide hormones are measured in pmol/L (Fig. 1).

**Fig. 2:**
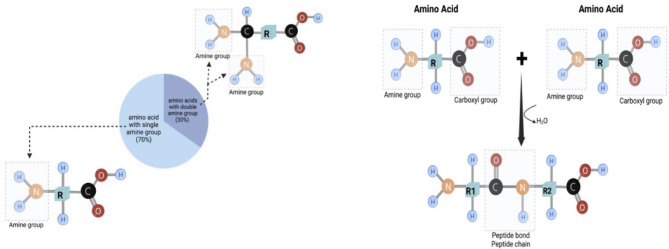
Schematic simplest representation of the placement of amine (NH_2_) groups on amino acids and peptides.

Moreover, it is crucial to keep in mind that each peptide, independently of its length, always has a minimum of one free amine group, thus mimicking a single amino acid with one amine group (Fig. 2). There are exceptions, approximately 30% of amino acids e.g., glutamine or arginine, have two amine groups, and can mimic two free amino acids. Thus, dipeptides composed of such amino acids can have 3 free amine groups, while tripeptides composed exclusively of glutamine can even have 4 free amine groups, and mimic respectively 3 or 4 free amino acids. Each of the amine groups in the ninhydrin reaction accounts for 1 free amino acid, while a single glutamine would be counted as 2 amino acids.

**Fig. 3:**
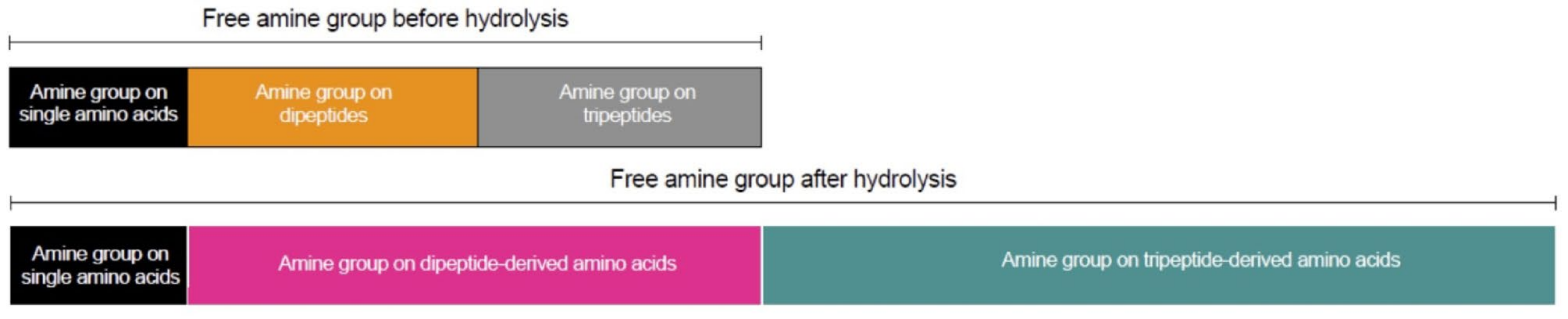
Schematic representation of the origin of amine groups in non-hydrolyzed and hydrolyzed plasma filtrate (10 kD filter).

Figure 3 highlights the source of the amine groups recognized by the ninhydrin method. Before hydrolysis, ninhydrin recognizes amine groups on amino acids, dipeptides or tripeptides. After hydrolysis, ninhydrin recognizes the amine groups on amino acids absorbed as free amino acids and on amino acids absorbed in the form of dipeptides or tripeptides and then reconstituted to free amino acids after hydrolysis.

The maximal ratio obtained using the revisited ninhydrin method, between Free Amine Groups after acidic (pH 5.5) Hydrolysis (FAGaH) to Free Amine Groups before acidic Hydrolysis (FAGbH), was 3 (factor W) (for detailed method description refer to^3^). Thus, dividing FAGbH by W we obtained the amount of amine groups on free amino acids appearing postprandially in the blood.

Based on assumptions from Fig. 3 we aimed to develop a simple algorithm to evaluate the ratio (percentage composition) of dipeptides, tripeptides and free amino acids which appear in the blood postprandially. To accomplish the above-mentioned aim, we had to construct a sensitive experimental model/design with alternating blood appearance of peptides and free amino acids. At first, we turned our attention to the digestive enzymes currently used in pancreatic enzyme replacement therapy (PERT) and their importance for protein digestion. Secondly, we decided to test the influence of the origin of dietary macronutrients and adaptation to them on the profile of the appearance of free amino acids versus peptides in the blood.

## Results

**Fig. 4:**
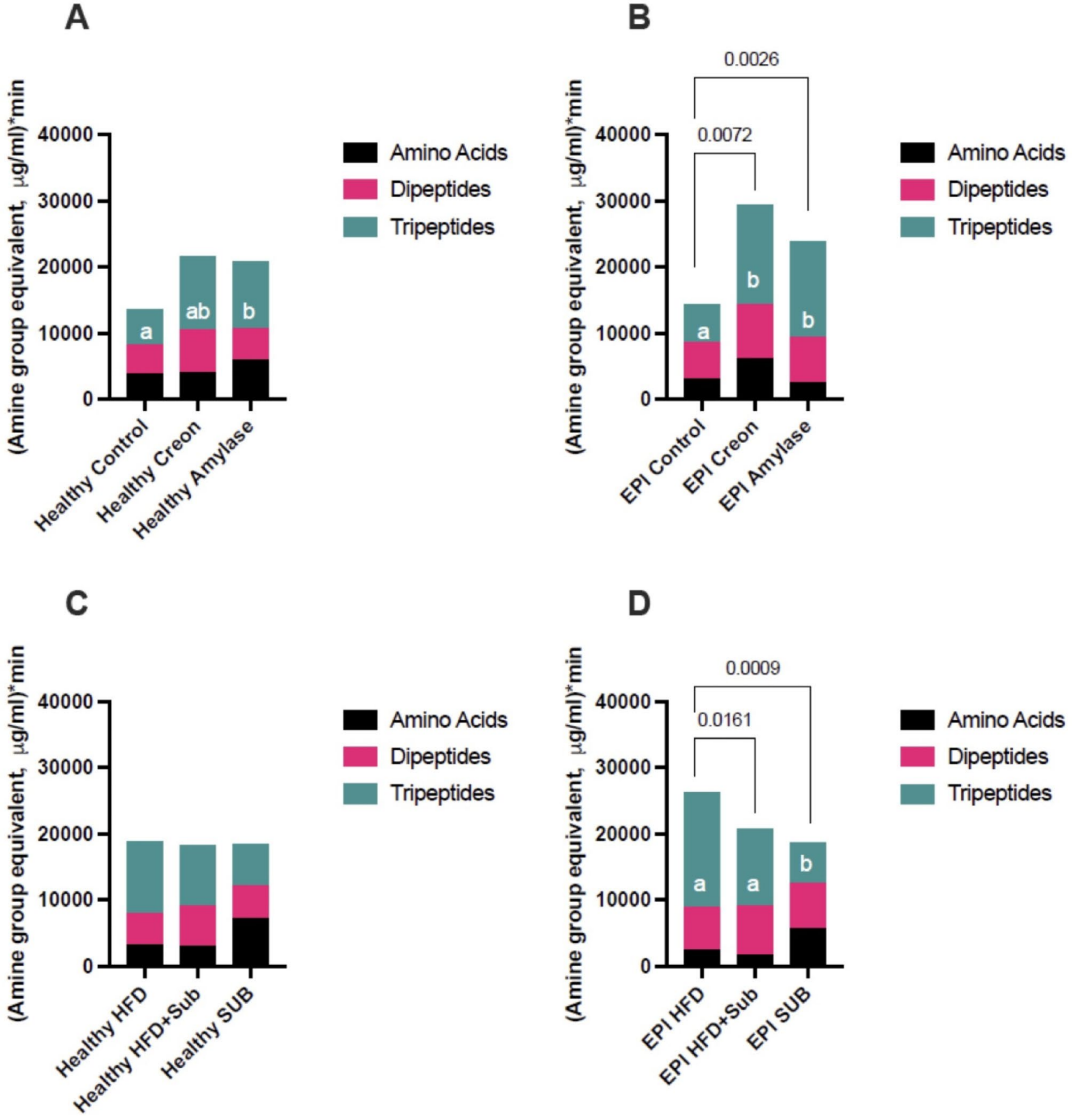
**A**–**D** Stacked bars plot of average AUC_T_ in equivalents of amine groups (µg/mL *min) of total amino acids. **A** Effect of enzyme supplementation (no treatment (Controls), treatment with Creon^®^ or Amylase) on Healthy pigs; **B** effect of enzyme supplementation (no treatment (Controls), treatment with Creon^®^ or Amylase) on EPI pigs; Effect of different MMTT compositions on Healthy (**C**) and EPI pigs (**D**). Different colors given within the bars show the most probable composition (absolute values) of the blood appearance of free amino acids (black) and amino acids derived from dipeptides (pink) and tripeptides (green). AUC_T_ is presented as the sum of AUCs for the free amino acids equivalent (AUC_F_), AUC for the dipeptide-derived amino acids equivalent (AUC_DD_) and AUC for the tripeptide-derived amino acids equivalent (AUC_TD_). Values are presented as Mean ± SD, differences were considered significant when *p* > 0.05, p values are given with the result bars. Different small letters written inside the result bars present significant differences for separate bar components. Any bar depicts 100% of amine groups after algorithm calculation.

**Fig. 5:**
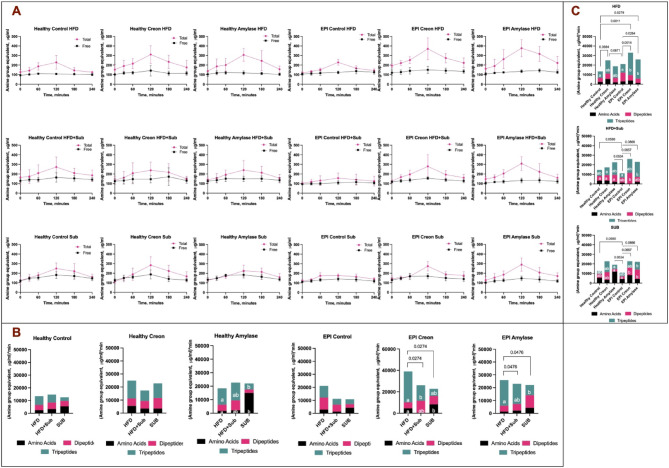
The appearance of amine group equivalents (corresponding to amino acids) in the blood postprandially shown as results from obtained with ninhydrin method (**A**) and calculated with algorithm (**B**, **C**). **A** Amine group equivalents (corresponding to amino acids) blood appearance curves during particular MMTT conducted on Healthy and EPI pigs treated with Creon or Amylase and different kinds of macronutrients; **B** vertically in columns summarised by algorithm—effects of diets on the amine group equivalents’ blood appearance, presented as AUC_T_ comparison (stacked bars); **C** horizontally in rows summarised by algorithm—effects of treatments on amine group equivalents’ blood appearance, presented as AUC_T_ comparison (stacked bars). Different colors given within the bars show the most probable composition (absolute values) of the blood appearance of free amino acids (black) and amino acids derived from dipeptides (pink) and tripeptides (green). AUC_T_ is presented as the sum of AUCs for the free amino acids equivalent (AUC_F_), AUC for the dipeptide-derived amino acids equivalent (AUC_DD_) and AUC for the tripeptide-derived amino acids equivalent (AUC_TD_). Values are presented as Mean ± SD, differences were considered significant when *p* > 0.05, p-values are given with the result bars. Different small letters written inside the result bars present significant differences for separate bar components. Any bar depicts 100% of amine groups after algorithm calculation.

The data presented as bar graphs are areas under the curves (AUCS) calculated using the algorithm from the results obtained with the revisited ninhydrin method (Figs. 4A–D, 5B, C). While data in Fig. 5A is presented as line graphs of original data of the so-called free amino acid amine groups before and total amino acid amine groups after peptide bonds hydrolysis. Algorithm calculated bars data in Fig. 5B correspond vertically to original data presented in columns in Fig. 5A. While algorithm calculated bars data in Fig. 5C correspond horizontally to original data presented in rows in Fig. 5A.

As shown in Fig. 4A–D (vertically), the blood appearance of free amino acids from dietary protein remained consistent between 15 and 20% of total amino acids. The remaining amino acids from dietary protein appear in the blood as either dipeptides (20–30% of the total amino acids pool) or tripeptides (30–65% of total amino acids pool), based on the algorithm estimation. Creon^®^, as well as alpha-amylase treatment, increased the blood appearance of tripeptide-derived amine groups, in both healthy and EPI pigs. Moreover, both Creon^®^ and alpha-amylase increased the total blood appearance of amino acids in EPI pigs, as represented by amine group equivalents (Fig. 4A, B). Adaptation to the HFD improved the blood appearance of tripeptide-derived amine groups during the HFD MMTT, compared to that observed during the SUB MMTT composed of new macronutrients to which the pigs were not adapted (Fig. 4D). The total blood appearance of amino acids was lower during both MMTTs with macronutrients to which the EPI pigs were not adapted or only partially adapted to (HFD + SUB and SUB MTT, Fig. 4D).

Figure 5A-C shows the levels of free amine groups and total amine groups in plasma samples obtained from Healthy and EPI pigs adapted to the HFD and to the different enzyme treatments (Creon^®^ vs. Amylase). MMTTs were performed on these groups either with the HFD to which animals were adapted, or with the HFD enriched with various substrates (HFD + SUB) to which the animals were only partially adapted, or the MMTT was performed with only the new macronutrients (MMTT with SUB).

The blood appearance of free amino acids (calculated by the algorithm) was low and often accounted for just < 10% of the total amino acids pool for the HFD (100% adaptation) and HFD + SUB (60% adaptation) MMTTs in both Healthy and EPI pigs. The blood appearance of dipeptides was around 30% of the total amino acids pool, with the remaining ca. 60% of the total amino acids pool constituted from tripeptides (Fig. 5C).

In both Healthy and EPI pigs, not adapted to the substrate components (SUB) of the MMTT, the percentage of free amino acids entering the blood reached an average of over 40% of the total amino acids pool, while the percentage of di- and tripeptides appearing in the peripheral circulation was drastically reduced (Fig. 5C). Interestingly, amylase supplementation resulted in an approximately 60% increase in the appearance of free amino acids in the blood of healthy animals. Statistical differences between amounts of di- and tripeptide-derived amino acids, as well as the blood appearance of free amino acids exhibited tremendous variability in relation to treatments.

**Fig. 6:**
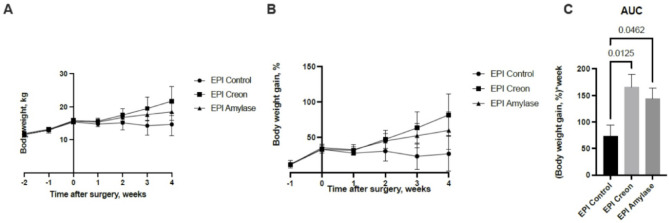
Body weight gain in pigs before and after pancreatic duct ligation (PDL). Data is expressed as Mean ± SD. The differences between the results were considered significant when *p* < 0.05. p values ranging between 0.1 and 0.05 were considered as a trend. Stars given with results describe significant differences in body weight gain on particular days of the experiment, between Control EPI pigs and EPI pigs treated with enzymes, when *p* < 0.05.

Figure 6 shows the weekly body weight gain of pigs from experimental groups throughout the experimental period. Control EPI pigs did not grow significantly during the entire experimental period. A significant increase in body weight gain and a similar growth pattern was observed in EPI pigs treated with either Creon^®^ or Amylase after PDL.

## Discussion

To test the sensitivity (applicability, reliability) of the updated ninhydrin method and the algorithm in distinguishing peptide absorption from free amino acids the MMTTs were performed on healthy animals and then again in the same animals when host pancreatic enzymes were eliminated with PDL. The different digestive enzymes (Creon vs. Amylase) and macronutrients used in the nutritional regime for pigs were used to introduce maximal variation and to test the sensitivity of the updated ninhydrin method and algorithm calculations. The updated ninhydrin method and algorithm allowed for the reliable estimation of the postprandial appearance of amine groups originating from either free amino acids or peptides and its variability in peripheral blood in the performed studies.

Use of the revisited ninhydrin method, supported by the described algorithm, provides results describing both the quality and quantity of postprandial peptides and free amino acids in the blood. Our studies, followed by the algorithm-based calculations, confirm that predominantly dipeptides and tripeptides appear in the blood postprandially, which was expected considering the presence of PepT1 transporters, responsible for dipeptide and tripeptide transportation, in the gut mucosa. At certain points, when massive appearance of peptides in the blood was noticed, the algorithm could reveal a scanty number of bigger peptides. Interestingly, expression of the afore-mentioned transporters is dependent on the presence of pancreatic enzymes in the gut lumen (especially amylase), as proven by Zaworski et al.^7^.

### Source (origin) of postprandial peptides in the blood

The origin of free amino acids appearing in the peripheral blood postprandially is rather obvious, however the source and type of peptides which appear in the blood is still poorly understood. An explanation for the postprandial appearance of peptides in the blood must involve their origin. To ensure a clear message for our readers, in the current paper we did not discuss the obvious role of the gut mucosa in the postprandial appearance of peptides and even their possible de novo synthesis in enterocytes followed by the exocytosis to the blood. Instead, we concentrated on the possible impact of gut enzymes and adaptation to dietary macronutrients on the postprandial appearance of peptides and free amino acids in the blood.

In addition to the regular digestive processes, performed by endopeptidases, exopeptidases and carboxypeptidases from the stomach and pancreas, which produce free amino acids and different kinds of peptides, the plastein rection in the gut lumen is also most likely involved^8,9^. The plastein reaction takes place in the presence of endopeptidases e.g., gastric pepsin is recognised as the primary non-specific enzyme responsible for extraordinary de-novo peptide formation from peptides and amino acids^8,9^. Trypsin and chymotrypsin, the main pancreatic specific endopeptidases, also participate in the plastein reaction^10–12^. It is important to note that during the plastein reaction, endopeptidases are not only involved in the cleaving of dietary proteins to oligopeptides, but also in the creation of new peptides from free amino acids, and in the modification of peptides derived through the classical action of endopeptidases on protein^13,14^. Importantly, the discussion concerning whether the quality of proteolysis and the blood appearance of its end products is dependent not only on the composition of enzymes, but also on the type of meal compounds and source of dietary protein, is still ongoing. *Nota bene*, the supplementation of plant-based protein feed for pigs and poultry (which become “vegans” by production technology) with huge numbers of essential amino acids, which has been normal practice for over 50 years^15,16^ probably results in the supplemented amino acids being used in the plastein reaction in the gut lumen to additionally improve the quality of plant proteins (making them more animal-like proteins), which in turn improves animal performance.

There are classical papers which state that dietary peptides do not appear in the blood as such, but in the form of amino acids, after being digested in the enterocytes^17^. On the other hand, there are papers postulating that close to 80% of dietary protein appears in the blood in the form of peptides, even longer than tripeptides^18^. Several previous studies, however, have confirmed that longer peptides are also absorbed from the gut^19^. In fact, with regards to the appearance of peptides in the blood postprandially and probably for host metabolism in general, it doesn’t really matter whether they come directly from the gut lumen or whether they are synthesized in the enterocytes. However, this question highlights a massive ‘gap’ in the current knowledge base with regards to the understanding of gut physiology.

Thus, the question arises as to the amount and size of dietary protein related peptides which appear in the blood postprandially. The appearance of peptides in the blood occurs as a result of the action of PepT1 transporters^20,21^. However, Rohm et al. showed that the transport of peptides, from the intestinal lumen to the blood, is an intensive process and cannot be exclusively accomplished via PepT1, thus, parallel mechanisms of peptide blood appearance must exist^22^. The reason why we are only considering dipeptides and tripeptides as nutritional is because only dipeptides and tripeptides have confirmed transport pathways from the gut (PepT1) and from the blood to the tissue cells (PepT2). The results of the current study support this finding. The value for factor W (ratio between total amine group equivalents after hydrolysis to amine group equivalents before hydrolysis) that we obtained was never higher than 3. This could serve as evidence that peptides of a maximal length of 3 amino acids (tripeptides) can be absorbed. It is interesting that PepT1 and PepT2 transporters are frequent in the kidney epithelium and protect peptides from escaping the body. However, dipeptides can be directly utilized by tissues as proven by Pierzynowski et al.^3^ (example of hair growth in Angora goats)^23^ and by other tissues as proven by Webb et al.^24^. Zhou et al. postulated that milk proteins are synthesized from free and peptide-bound amino acids^25^.

In terms of overall metabolism, as well as the regulation of blood peptide appearance, one should bear in mind the extremely important role of the brush border amino peptidases. Holst et al.^26^ showed the importance of these peptidases in the regulation of GLP1 blood appearance. It has also been shown that amino peptidases improve the taste of a meal^27^ and various features of the skin^28^. One can speculate that brush border amino peptidases regulate the entrance of gut peptide hormones into the blood^26^ after their paracrine action. One can hypothesize that amino peptidases could also regulate the number and type of peptides absorbed, specifically dipeptides and tripeptides, out of 400 and 8000 dipeptides and tripeptides respectively, that are theoretically available. It must be emphasized that longer dietary peptides as protein derivatives could easily act as hormones and thus it could be terribly dangerous if they were absorbed. E.g., theoretically, the blood appearance of the biologically active tetrapeptide CCK-4 (Trp-Met-Asp-Phe), derived from food, is possible. CCK4 causes a deep panic effect, even in very small, (*pmol)* concentrations when infused iv, as postulated by Rehfeld^29^. Thus, the possible blood appearance of peptides longer than triamino acid peptides must be carefully explored. One should consider that such peptides could jeopardize metabolism and act directly against the biological integrity of the organism in certain creatures. Thus, future studies should investigate how the appearance of blood peptides, both those originating from food protein and those originating from the gut lumen, can be regulated.

### Algorithm calculation

The ratio 1 < W < 3 between total and free amine groups measured by the ninhydrin method covers the most probable percentage of absorbed amino acids, dipeptides, tripeptides and only a scanty amount (if any at all) of longer peptides. AUCs of the blood appearance of total amine group equivalents, calculated as the area under the curve restricted by the baseline for free amine groups, provides a visual representation of the free amino acids- and peptide-derived amine groups, originating from dietary protein and entering the peripheral blood postprandially. The biggest limitation for the calculation performed by the arbitrary choosen for simplicity the lineal algorithm is the assumption that all possible proportions of free amino acids and di and tripeptides are possible in their appearance in the blood. Aperance longer peptides is terminally limited and can be neglected in the calculations. Moreover from a biological perspective, we know that such apearence can be dangerous and most probably is not true. Thus, the algorithm we developed for calculation of the proportion of free amino acids and specific nutritional peptides must be further evaluated in a series of in vitro and in vivo experiments, to set up the most probable not linear proportions for particular animal species, age, training, metabolic status, etc. The ratios achieved in these types of experiments are dependent on multiple factors, which probably do not have an equal distribution. Thus, the actual statistical comparison of the number of amino acids derived from dipeptides or tripeptides should be treated as an example of the counting possibility of the suggested algorithm for the moment.

### Evaluation of the study on animals

The results obtained from the present study prove that the use of an animal model with varying gut enzymology and dietary components can drastically change the proportions of dietary dipeptides, tripeptides and free amino acids entering the peripheral blood postprandially. Moreover, the results highlight that dietary dipeptides and tripeptides are the main products of protein digestion. Thus, the peptides which appear in the blood postprandially constitute the majority of amino acids in comparison to the portion represented by free amino acids. The above statements can be clearly recognised on the complex Fig. 5A–C.

The existence of two complementary, specific transporters, PepT1 which transports peptides from the gut to the circulation and PepT2 which transports peptides from the blood to specific tissues, in some way highlights the possible benefits of the transport of absorbed peptides from the gut lumen to the tissues. The results obtained also prove that PERT enzymes are indispensable, crucial factors in the blood appearance of nutritional dietary peptides.

In the current study, both Healthy and EPI pigs were kept on full portion mono-diet, which means that similarly to that which occurs in regular pig production, the pigs received the same diet for each meal. Our MMTTs, with the HFD or with the HFD + SUB, were performed on animals that were either fully adapted or partially adapted to the diet. The MMTT with the SUB was performed without prior adaptation. Thus, it is interesting that the blood appearance of free amino acids during the SUB MMTT, as calculated by the algorithm, was higher than that observed during the other MMTTs and achieved up to over 60% of total amino acids appearing in the blood. During the MMTTs to which the pigs were either fully (HFD) or partially adapted (HFD + SUB), the blood appearance of free amino acids was around 10% of total blood appearance of amino acids.

It is also interesting that both EPI groups that received feed supplemented with enzymes (Creon^®^ or Amylase) displayed better growth than EPI pigs without enzyme supplementation. The improved growth was obviously expected following Creon^®^ supplementation, but it was not ‘a given’ following Amylase supplementation. We attribute the improved growth following enzyme supplementation to increased PepT1 expression in the gut in both the Creon^®^ and Amylase groups^7^which coincided with improved peptide blood appearance in the actual experiment. It is also worth stating that the amino acids derived from tripeptides formed the majority of the amino acids appearing in the peripheral blood postprandially.

In summary, the algorithm we developed and used to evaluate the results obtained with the revisited ninhydrin method, made it possible, for the first time, to estimate with a high level of probability, the percentage of nutritional dipeptides, tripeptides and free amino acids’ postprandial appearance in the peripheral blood (as dietary protein digestion end-products). The animal and dietary models chosen are sensitive and effective enough to highlight the role of pancreatic enzymes (Creon^®^ and Amylase) and feed composition in the postprandial appearance of peptides and free amino acids in the peripheral blood. Enzymes essentially improve the postprandial appearance of dipeptides and tripeptides in the blood of Healthy and EPI pigs. Lack of dietary adaptation to macronutrients used ad hock in the MMTT provokes an essential increase in the postprandial appearance of free amino acids in the blood.

### Paper significance

Protein digestion and subsequent peptide appearance in the blood have not been studied in depth. A simple mathematical model/algorithm was developed to evaluate the effectiveness of a revisited ninhydrin method to measure postprandial appearance of amine groups derived either from free amino acids or peptides in the blood. To ensure maximal variability of peptides and free amino acids appearing in the blood, we used macronutrients of different origins in the diet of healthy and exocrine pancreas insufficient (EPI) pigs.

The results calculated by the algorithm showed that the postprandial appearance of amine groups from di- and tripeptides significantly exceeded those derived from free amino acids. Enzymes added to the pigs’ feed increased the appearance of peptide-derived amine groups in the blood. Surprisingly, amylase feed supplementation in healthy animals during the mixed meal tolerance test (MMTT) with exclusively new macronutrients, to which the pigs were not adapted, provoked a massive increase in the postprandial appearance of amine groups originating from free amino acids in the peripheral blood. The ratio of di- and tripeptide-derived amine groups appearing postprandially in the blood was affected by both enzyme supplementation and macronutrients used in the MMTT.

The old, revisited ninhydrin method and new algorithm provide valuable insight on appearance of digested protein products in the blood. Peptides—free amino acids ratio in the blood could be important considering the definition of biological age. E.g., blood appearance of peptides in young animals is attributed to the high frequency of PepT1 in gut mucosa, which disappears with age. The way in which dietary proteins appear in blood as products of digestion, in the form of free amino acids and peptides, lead us to thinking about how these proportions affect metabolism. Undoubtedly, these proportions can be of importance in both a healthy state (sportsman, body builders etc.) and during metabolic, systemic, genetic, etc. sicknesses. The quality of meal proteins (e.g., new biotechnological proteins) and dietary habits (e.g., vegans) can also affect the profile of amino acids and peptides entering the circulation. Finally, the coincidence of the presence of PepT1 in the gut lumen and PepT2 in peripheral tissues (both are responsible for di- and tripeptide transport) opens the door for future studies on the metabolic role of postprandial nutritional di-and tripeptides.

## Materials and methods

### Estimation of the amount of amine groups in the blood

The ninhydrin method modified by Pierzynowska et al.^3^ was used for the measurement of the appearance of free amino acids versus peptides in the blood. In short, large proteins were eliminated from plasma using 10 kDa-cut-off centrifugal filters. Free and total amino acid-derived amine groups were measured in the permeate before and after its hydrolysis. Peptide-derived amine groups were used as a measurement of respective amino acids and quantified using the suggested algorithm.

### Algorithm

For estimation of the ratio of dipeptides, tripeptides and free amino acids, which appear in circulation postprandially, we developed the following algorithm:

The ratio between optical density readings of hydrolyzed filtrate (FAGbH), and in intact filtrate (FAGaH) was named W.

T—equivalent of total number of amine groups in hydrolyzed filtrate.

F—equivalent of free amine groups in non-hydrolyzed filtrate.

$${\text{Factor W}}={\text{T }}:{\text{ F }}\left( {{\text{the quotient of T and F}}} \right)$$

Each amine group corresponds to one free amino acid or, more often, one dipeptide or tripeptide with the restrictions given below.

To answer the question on the average ratio of amino acids and peptides, it is necessary to use a more formal language. For this purpose, we will introduce the following notations and assumptions:

W = T/F, W ≥ 1, Δ = T − F=(W − 1)F and F = 1 (this is how we normalize for the purposes of considerations).

a-number of amino acids (it is assumed that they possess only one amine group), x = (x_1_ + x_2_ + x_3_) ≥ 0 - number of dipeptides, where x_i_ (i = 1,2,3) denotes the number of peptides consisting of 1, 2 or 3 amine groups, respectively,

y=(y_1_ + y_2_ + y_3_+y_4_) ≥ 0 - number of tripeptides, where y_i_ (i = 1,2,3,4) denotes the number of peptides consisting of 1, 2, 3 or 4 amine groups, respectively.

We assume that the number of other peptides is small enough to omit them in the following considerations.

After hydrolysis, dipeptides - x_i_ (i = 1,2,3) yield i + 1 amine group, and tripeptides - y_i_ (i = 1,2,3,4) yield i + 2 amine groups, respectively.

The symbols a*, x* and y* denote the share of the estimated amine groups (before hydrolysis) by amino acids and peptides a, x and y. After hydrolysis, the share of the signaling amine groups are designated by the symbols a′, x′ and y′, respectively. Therefore, (a*, x*, y*) and (a′, x′, y′) denote the structures F and T, respectively.

We also assume the following simplifying constraints:

1 $${\text{x}}_{1}=({1} - {\text{a}}){\text{x}},\quad {\text{y}}_{1}=({1} - {\text{b}}){\text{y}},\quad {\text{where}}\quad 0 \leqslant {\text{a}} \leqslant 0.3,\quad 0 \leqslant {\text{b}} \leqslant 0.3 \quad {\text{and}}\quad {\text{x}}_{2} ={\text{x}}_{3},\quad {\text{y}}_{2}= {\text{y}}_{3}= {\text{y}}_{4}$$

With the above assumptions, we have the following two relations:

2 $${\text{a}}+{\text{x}}_{1}+{\text{2x}}_{2}+{\text{3x}}_{3}+{\text{y}}_{1}+{\text{2y}}_{2}+{\text{3y}}_{3}+{\text{4y}}_{4}={\text{F}}= 1$$

3 $${\text{D}}={\text{T}} - {\text{F}}={\text{x}}+{\text{2y}}={\text{W}} - {\text{1}}.$$

Based on (1), (2), (3) we can construct the following formulas:

4 $${\text{y}}=({\text{W}} - {1} - {\text{x}})/{\text{2 and x}}=[{1} - {\text{a}} - ({1}+{\text{2b}})({\text{W}} - {1})/{2}]/[0.5 + 1.{\text{5a}} - {\text{b}}]$$

That is, both the value of x and y can be calculated given the values of a, α, β and W.

Since x and y are non-negative values, it can be easily shown that

$${\text{Max }}[1 - (1+1.{\text{5a}})\left( {{\text{W}} - 1} \right);0] \leqslant {\text{a}} \leqslant 1 - (1+{\text{2b}})\left( {{\text{W}} - 1} \right)/2.$$

Therefore, for a fixed value of the index W, it is known that (α, β, a) are elements of the set:

5 $$\left[ {0;0.{\text{3}}} \right] \times {\text{ }}\left[ {0;0.{\text{3}}} \right]{\text{ }} \times \left[ {{\text{Max}}} \right[{\text{1}} - ({\text{1}}+{\text{1}}.{\text{5a}})\left( {{\text{W}} - {\text{1}}} \right);0];{\text{1}} - ({\text{1}}+{\text{2b}})\left( {{\text{W}} - {\text{1}}} \right)/{\text{2}}].$$

In a case where we have no other information, other than the value of the W index, we should make a conservative assumption that all points in the set defined by formula (5) are equally probable (important). With this assumption, it is relatively easy to calculate the average value of a^*^ for a fixed value of W. Using the calculated mean value of a* and using formulas (1) and (4), we can calculate the mean structure (a*, x*, y*) for F. Using the average structure (a*, x*, y*) for F, formula (4) and the fact that after hydrolysis we gain x (from dipeptides) and 2y (from tripeptides) of signaling amine groups, we can easily calculate the average structure (a′, x′, y′) for T at a fixed value of W.

### Animals

The present study was performed in strict accordance with the recommendations in the Guide for the Care and Use of Laboratory Animals of the National Institutes of Health and reported in accordance with the ARRIVE guidelines. All efforts were made to minimize animal suffering. The study was approved by the Second Local Ethics Committee for Animal Experimentation in Warsaw, Poland (approval no. WAW2/025/2022). The experiment was performed on 18 crossbred ((Polish Landrace × Yorkshire) × Hampshire)) pigs (*Sus scrofa domesticus*), purchased from a local herd (Karniewek, Poland), a 50:50% mix of both genders (males were castrated on day 3 of their life), with a body weight of 15 ± 2.3 kg at the beginning of the study.

### Experimental design

**Fig. 7:**
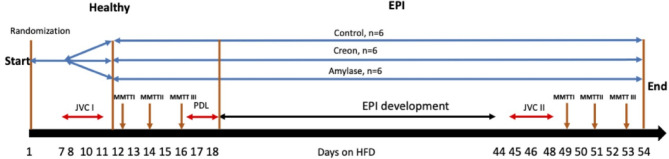
Experimental design and study treatments. *JVC* jugular vein catheterization surgery; *MMTT* mixed meal tolerance tests (adapted MMTTI − HFD, acute MMTTII − HFD + SUB, acute MMTTIII − SUB); *SUB* substrates, *PDL* pancreatic duct ligation surgery, *EPI* exocrine pancreatic insufficiency.

From the first day all pigs were randomised to either the Control, Creon or Amylase group and adapted to the high fat diet (HFD) (Fig. 7). Additionally, animals from the Creon group and Amylase group were adapted to Creon^®^ or microbial Amylase supplementation during morning and evening meals (Fig. 7). All pigs underwent jugular vein catheter implantation on days 8 and 9 ^30^. Starting from day 12, and every second day thereafter, the respective mixed meal tolerance tests (MMTTs) were performed on healthy pigs (Fig. 7). On days 17 and 18, all pigs were subjected to pancreatic duct ligation (PDL) surgery^30,31^. After EPI development (3 weeks from PDL), on days 45 and 46, complementary jugular vein catheters (JVC II) were implanted. Starting from day 49, and every second day thereafter, identical MMTTs, with identical treatment and experimental order as those performed on healthy pigs, were performed on EPI pigs.

### Feed

During the study pigs were adapted to the HFD (Kcynia, Morawski Plant, Poland; 18 g cereal protein, 20 g rapes oil, 50 g starch cereals, 3 g crude fiber, 4 g ash, 5 g water) which they were fed in an amount equivalent to 4% of their body weight daily, with 1% given at the morning meal (09:00–10:00 h) and 3% at the afternoon meal (17:00–18:00). Upon arrival at the experimental unit, the pigs were fed a cereal-based, pelleted, standard diet and abruptly changed to a HFD on the first day of the adaptation period (Fig. 7).

### Selected substrates for acute MMTT

The following substances were chosen to create the acute test meal used for the MMTTs, to mimic a standard high-fat meal (Table 1): docosahexaenoic acid (DHA) and eicosapentaenoic acid (EPA)—Kinoko Life 2000 mg Omega-3 softgels; 500 mg EPA and 250 mg DHA per capsule, Whey—California Gold Nutrition 100% Whey Protein Isolate, Unflavoured (www.californiagoldnutrition.com), Potato starch—Roots Circle Gluten-Free Potato Starch (https://www.amazon.com).

**Table 1:** Composition of mixed meal tolerance tests (MMTT) offered as morning meals.

	MMTT I HFD	MMTT II HFD + SUB	MMTT II SUB
High Fat Diet Components	28 g cereal protein + 32 g fat - rapeseed oil + 79 g starch cereals + 6 g crude fiber + 8 g ash + 7 g water = 160 g	18 g cereal protein+ 20 g rapeseed oil + 50 g starch cereals + 3 g crude fiber+ 4 g ash + 5 g water = 100 g	0
Substrate (SUB) Components	0	4 g DHA/EPA + 10 g whey + 32 g potato starch + 8 g soya oil 3 g ash + 3 g water = 60 g	4 g DHA/EPA + 28 g whey + 85 g potato starch + 28 g soya oil 8 g ash + 7 g water = 160 g

Morning meal accounted for 1% of the daily requirements of pigs weighing approximately 16 kg. The meals were isoenergetic. Between MMTTs, pigs were fed the HFD – 1% and 3% respectively for morning and evening meals.

### Enzymes

Amylase DS100 (Amano Enzymes, *Aspergillus oryzae*, 4000 Units/dose) or Creon^®^ 25,000 (Abbot USA, 100,000 lipase units/dose), were administered to pigs with the morning and evening meals, depending on experimental design randomization. Control Group (*n* = 6) was fed HFD alone, Creon Group (*n* = 6) was fed HFD + Creon^®^ (2 × 100,000 units daily), Amylase group was fed HFD + Amylase (2 × 4000 units daily) at the morning and evening meals, respectively, for the entire duration of the experiment. Detailed study design is shown in Fig. 7.

### Blood sampling

Blood samples were collected during the MMTT via a jugular vein catheter, at one hour and then again at one minute prior to feeding and then at 5, 15, 30, 45, 60, and 120 min after feeding and transferred to BD Vacutainer^®^ glass Aprotinine K_3_EDTA tubes (BD Diagnostics, New Jersey, USA). The blood samples were immediately placed on ice before they were centrifuged at 3000×*g* for 15 min at 4 °C, and plasma was separated and stored at – 80 °C until further analysis. Blood glucose concentrations were measured directly following blood sampling using a glucometer and test strips (Accu-Chek Aviva, Roche Diagnostics, Germany).

### Statistical analysis

Statistical analysis was performed on the data obtained from this study using an ordinary one-way ANOVA for normally distributed datasets or Kruskall–Wallis test when data was not normally distributed. The data distribution was assessed using the Shapiro-Wilk normality test. Outliers within data sets were identified using the ROUT method of regression (Q = 0.05%). All the analyses were carried out using GraphPad Prism 10.0, San Diego, USA. Data was not corrected for multiple comparisons. Differences were considered significant if *p* ≤ 0.05; differences were considered as a trend when *p* ≤ 0.1; data with Gaussian distribution is expressed as mean ± standard deviation (SD), data with non-Gaussian distribution is expressed as median ± intraquartile range (IQR). Area under the curve (AUC) data is baseline adjusted. AUC for the equivalent of total number of amine groups in hydrolysed filtrate (AUC_T_) was considered to consist of AUC for the free amino acids equivalent (AUC_F_), AUC for the dipeptide-derived amino acids equivalent (AUC_DD_) and AUC for the tripeptide-derived amino acids equivalent (AUC_TD_).

## Data Availability

The raw data supporting the conclusions of this article will be made available by the authors upon request. Data requests should be addressed to Dr. Kateryna Pierzynowska (Kateryna.Pierzynowska@biol.lu.se).
